# Late diagnosis of CKD and associated survival after initiation of renal replacement therapy in Kazakhstan: analysis of nationwide electronic healthcare registry 2014–2019

**DOI:** 10.1080/0886022X.2024.2398182

**Published:** 2024-09-04

**Authors:** Valdemir Kim, Gulnur Zhakhina, Arnur Gusmanov, Yesbolat Sakko, Mariyam Kim, Meruyert Madikenova, Zhannat Kuanshaliyeva, Alpamys Issanov, Ainur Assan, Marina Khvan, Altay Nabiyev, Sholpan Altynova, Abduzhappar Gaipov

**Affiliations:** a Department of Medicine, School of Medicine, Nazarbayev University, Astana, Kazakhstan; b Clinical Academic Department of Internal Medicine, CF “University Medical Center”, Astana, Kazakhstan; c School of Population and Public Health, University of British Columbia, Vancouver, Canada; d Department of Medicine, Khoja Akhmet Yassawi International Kazakh-Turkish University, Turkestan, Kazakhstan; e Department of Medical and Regulatory Affairs, CF “University Medical Center”, Astana, Kazakhstan

**Keywords:** Chronic kidney disease, renal replacement therapy, late diagnosis, survival prognosis, end-stage renal disease

## Abstract

Chronic kidney disease (CKD) presents a significant global health challenge, often progressing to end-stage renal disease (ESRD) necessitating renal replacement therapy (RRT). Late referral (LR) to nephrologists before RRT initiation is linked with adverse outcomes. However, data on CKD diagnosis and survival post-RRT initiation in Kazakhstan remain limited. This study aims to investigate the impact of late CKD diagnosis on survival prognosis after RRT initiation. Data were acquired from the Unified National Electronic Health System (UNEHS) for CKD patients initiating RRT between 2014 and 2019. Survival post-RRT initiation was assessed using the Cox Proportional Hazards Model. Totally, 211,655 CKD patients were registered in the UNEHS databases and 9,097 (4.3%) needed RRT. The most prevalent age group among RRT patients is 45–64 years, with a higher proportion of males (56%) and Kazakh ethnicity (64%). Seventy-four percent of patients were diagnosed late. The median follow-up time was 537 (IQR: 166–1101) days. Late diagnosis correlated with worse survival (HR = 1.18, *p* < 0.001). Common comorbidities among RRT patients include hypertension (47%), diabetes (21%), and cardiovascular diseases (26%). The history of transplantation significantly influenced survival. Regional disparities in survival probabilities were observed, highlighting the need for collaborative efforts in healthcare delivery. This study underscores the substantial burden of CKD in Kazakhstan, with a majority of patients diagnosed late. Early detection strategies and timely kidney transplantation emerge as crucial interventions to enhance survival outcomes.

## Introduction

Chronic kidney disease (CKD) poses a global health concern, estimated at a prevalence of 9.1% [1]. Over time, CKD advances to end-stage renal disease (ESRD), necessitating life-saving renal replacement therapy (RRT). While ESRD incidence and prevalence are increasing in Kazakhstan [2], understanding prognosis factors for this patient group remains limited [3], with late referral (LR) being one such factor.

LR is defined as the first nephrologist encounter within 1–6 months before RRT initiation [4], and an ultra-late referral (ULR) refers to referral less than 30 days before RRT initiation [5]. Approximately 30% of patients globally present with LR and start RRT nonelectively, ranging from 22% to 71.6% [6]. Causes of LR are categorized into avoidable and unavoidable. Avoidable cases include patients with slowly progressive CKD known to primary care physicians and specialists, while unavoidable cases involve patients with an unknown CKD course [7]. Reasons for LR include asymptomatic CKD progression, lack of awareness about disease severity, pre-ESRD care importance, communication gaps, and concerns about patient loss or disturbing families [8].

Consequences of LR include severe hematological, ­endocrine, and metabolic abnormalities at presentation, such as hypoalbuminemia, anemia, hyperparathyroidism, hypocalcemia, hyperphosphatemia, hypertension, and heart failure. Some of these abnormalities are associated with worse survival outcomes post-RRT initiation [9]. Patients referred late experience longer hospitalizations and higher costs [10,11]. LR also impacts vascular access and transplantation chances [12,13]. For instance, patients managed by nephrologists had a lower need for temporary venous catheterization [14].

Studies show conflicting results regarding long-term survival differences between early referral (ER) and LR groups. One study found no difference in five-year survival between ER and LR groups, despite higher short-term morbidity in the LR group [15]. Conversely, a systematic review indicated significant mortality reduction for ER patients [16]. Another study showed no significant difference in 1-year mortality rates between LR and ER groups despite a decrease in LR percentage and improved initial conditions [17].

Kazakhstan faces challenges in early CKD diagnosis and timely referral due to its vast territory and limited access to qualified healthcare. While some countries advocate for renal monitoring among individuals on nephrotoxic medications [18], Kazakhstan typically diagnoses CKD in symptomatic patients or those with comorbidities [19]. A recent study found CKD prevalence exceeded 38,000 cases per million population in 2020, projected to triple by 2030 [20]. Although ESRD prevalence and incidence are growing in Kazakhstan [3], studies on LR and its effect on survival post-RRT initiation are lacking. This study aims to evaluate various factors, including late CKD diagnosis, on patients’ survival prognosis after RRT initiation in Kazakhstan.

## Materials and methods

### Preparation of the dataset

Data were acquired from the Unified National Electronic Health System (UNEHS), identifying a total of 211,655 CKD patients, of which 9,097 (4.3%) received RRT, including dialysis or kidney transplantation. To assess the impact of various independent variables on survival post-RRT initiation, the Cox Proportional Hazards Model was employed, with the outcome event being death or alive status by the end of observation period.

The study was approved by the Institutional Review Ethics Committee of Nazarbayev University (NU-IREC 490/18112021), with exemption from informed consent.

Patient information was sourced exclusively from UNEHS, specifically from the Electronic Outpatient Registry (EOR), Electronic Inpatient Registry (EIR), and Registry of Chronic Kidney Disease (RCKD) [21]. The RCKD, which contains the majority of information regarding patients undergoing RRT, has limited availability until 6th of April 2019. Therefore, the final observation endpoint was set by this day. The study was approved by the Institutional Review Ethics Committee of Nazarbayev University (NU-IREC 490/18112021), with exemption from informed consent.

The data preparation process involved several key steps. First, the registries were assembled by consolidating pieces divided by years (2014–2019) and regions of Kazakhstan. Patient identification for CKD and associated diagnosis dates was achieved using ICD-10 CM Diagnosis codes (Supplementary Table 1). ICD-10 codes corresponding to acute kidney injury (AKI) (N17) were excluded. Comorbidities and their diagnosis dates were identified in a similar manner, initially represented as duplicate observations for each patient. Dialysis or transplantation status and associated dates were determined using ICD-10 CM Diagnosis codes and ICD-9 CM Procedure codes (Supplementary Table 1).

Further steps included the translation of variables, deducing regions from addresses, appending all three registries using a combination of date of birth and RPN ID (population registry identification number), and combining information about the main diagnosis, comorbidities, and RRT status with associated dates. The earliest diagnosis dates among the three registries were used for each diagnosis and comorbidity. For data analysis, patients with the earliest diagnosis dates between January 1st, 2014, and April 6th, 2019, were considered, with data beyond April 6th, 2019, replaced with **‘**NA’ to maintain consistency.

**Figure 1.**
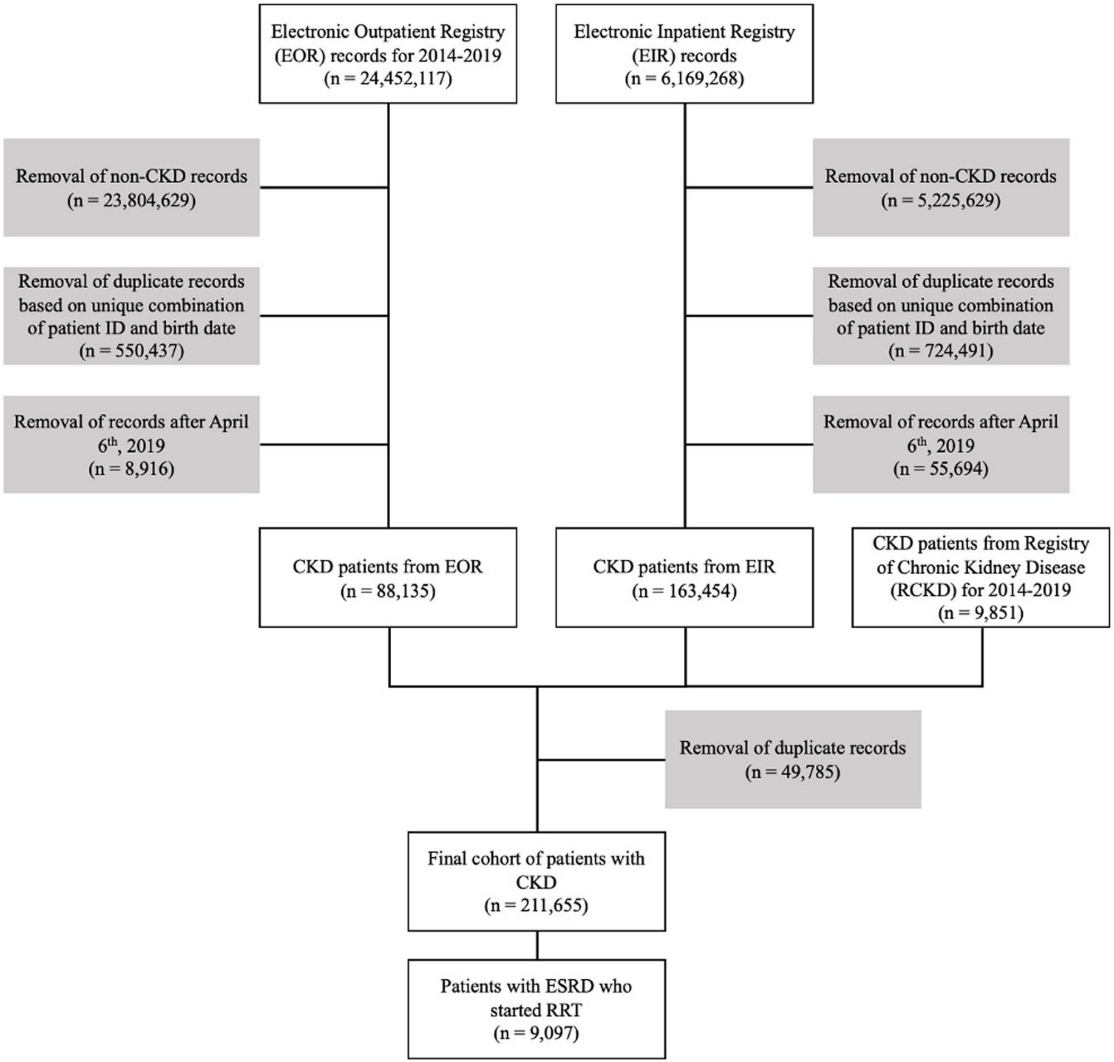
Flow chart of cohort set-up.

The Electronic Outpatient Registry (EOR) provided information about 88,135 CKD patients, the Electronic Inpatient Registry (EIR) had data on 163,454 CKD patients, and the Registry of Chronic Kidney Disease (RCKD) contributed information on 9,851 CKD patients. After merging all three datasets based on date of birth and RPN ID, the final dataset comprised 211,655 unique patient observations. The flowchart illustrating the data preparation process is depicted in Figure 1.

Codes utilized for the identification of patients with CKD and other comorbidities were retrieved from the USRDS 2021 Annual Data Report, specifically from the Analytical Methods section. Both adult and pediatric CKD codes were applied since our registries contained all age groups. Additionally, dialysis or transplantation-related ICD-10 CM Diagnosis codes were utilized. These codes are summarized in Supplementary Table 1.

### Exposures and covariates

Basic patient characteristics, encompassing age group, year of diagnosis, sex, and ethnicity, were delineated for the entire CKD cohort (*n* = 211,655) and the subgroup of patients who initiated RRT (*n* = 9,097). The latter group was further categorized into those commencing RRT within 6 months from the earliest CKD diagnosis, defined as an early diagnosis (ED), and those initiating RRT after 6 months from the earliest CKD diagnosis, defined as a late diagnosis (LD). Details such as age groups, year of diagnosis, sex distribution, ethnicity distribution, and comorbidities (hypertension (HTN), diabetes mellitus (DM), glomerulonephritis (GN), cystic disease, heart failure (HF), atherosclerotic heart disease (ASHD), dysrhythmia, cerebrovascular accident/transient ischemic attack (CVA/TIA), peripheral vascular disease (PVD), chronic obstructive pulmonary disease (COPD), liver disease, and cancer) were described. Additionally, the median time of follow-up, median time from diagnosis to the initiation of RRT, transplantation history, and the total number of deaths from 2014 to April 6th, 2019, were included in the characterization.

### Outcome of interest

The outcome of interest was all-cause mortality after initiation of RRT between ED and LD groups. The follow-up period commenced at the initiation of RRT until death or April 6th, 2019.

This study adheres to the Strengthening the Reporting of Observational Studies in Epidemiology (STROBE) guidelines for reporting observational studies [22]. The STROBE checklist was used to ensure comprehensive and transparent reporting of the study’s methodology and findings.

#### Statistical analysis

Data are summarized as percentages for categorical variables and as mean (standard deviation) or median (quartile 1–3), as appropriate. Categorical variables were compared using c2 tests. Continuous variables were compared using *t*-tests, Mann–Whitney U-tests, or analysis of variance, as appropriate.

R version 4.1.2, coupled with R Studio version 2022.02.0 + 443, facilitated data management. The process involved assembling and cleaning the data, utilizing the ‘tidyverse’ package for preparation, and cleaning. The subsequent step involved survival analysis, employing the ‘survminer’ and ‘survival’ packages. Finally, Microsoft Excel and the ‘ggplot2’ package were utilized for data visualization.

The Kaplan–Meier Survival Analysis (KMSA) was utilized to compare the survival probability after the initiation of RRT between LD and ED patients. KMSA survival curves were generated, and a table presenting numerical values of survival probability at various time points was constructed.

For multivariate survival analysis, the Cox Proportional Hazards Model was employed. This involved assessing several variables (age, sex, ethnicity, region, late diagnosis status, comorbidity score, and transplantation status) simultaneously to determine their collective impact on survival. The findings were presented through a forest plot, illustrating the associated hazards ratios (HRs) for each variable.

The comorbidity score was calculated using the formula: 1*(DM + ASHD) + 2*(CVA/TIA + PVD + COPD + Dysrhythmia + Liver Disease + Cancer) + 3*HF [23]. Specifically designed for ESRD patients on dialysis, this comorbidity score aids in considering potential confounders associated with comorbidities during survival analysis.

## Results

### Overall characteristics of ESRD patients

Table 1 summarizes the characteristics of patients who initiated RRT from the Unified National Electronic Health System (UNEHS). The total number of this patient population is 9,097. Among those, 74% were diagnosed with CKD and started RRT within a 6-month interval, categorized as a late diagnosis.

**Table 1.** Characteristics of patients who started RRT.

	Patients who started RRT			
	All patients	LD: < 6 months from diagnosis	ED: ≥ 6 months from diagnosis	
	*n* = 9,097 (100)	*n* = 6,699 (74)	*n* = 2,398 (26)	*p*-value
**Age, years**				< 0.001
0–17	280 (3.1)	177 (2.6)	103 (4.3)	
18–44	2,929 (32)	2,152 (32)	777 (32)	
45–64	4,150 (46)	2,970 (44)	1,180 (49)	
65–74	1,306 (14)	1,028 (15)	278 (12)	
75+	433 (4.8)	373 (5.6)	60 (2.5)	
**Median age, years [IQR]**	52 [37-62]	53 [38-63]	51 [36-60]	
**Year of diagnosis**				< 0.001
2014	2,276 (25)	1,414 (21)	862 (36)	
2015	1,849 (20)	1,263 (19)	586 (24)	
2016	1,749 (19)	1,268 (19)	481 (20)	
2017	1,592 (18)	1,241 (19)	351 (15)	
2018	1,366 (15)	1,248 (19)	118 (4.9)	
2019*	266 (2.9)	266 (4.0)	0 (0)	
**Sex**				< 0.001
Males	5,064 (56)	3,801 (57)	1,263 (53)	
Females	4,034 (44)	2,899 (43)	1,135 (47)	
**Ethnicity**				< 0.001
Kazakh	5,796 (64)	4,098 (61)	1,698 (71)	
Russian	1,513 (17)	1,201 (18)	312 (13)	
Uzbek	316 (3.5)	230 (3.4)	86 (3.6)	
Ukrainian	242 (2.7)	188 (2.8)	54 (2.3)	
Uyghur	247 (2.7)	186 (2.8)	61 (2.5)	
Korean	204 (2.2)	162 (2.4)	42 (1.8)	
Other	780 (8.6)	635 (9.5)	145 (6.0)	
**Comorbidities/causes**				< 0.001
HTN	4,309 (47)	2,847 (42)	1,462 (61)	
DM	1,920 (21)	1,209 (18)	711 (30)	
GN	1,801 (20)	928 (14)	873 (36)	
Cystic disease	280 (3.1)	155 (2.3)	125 (5.2)	
CVD	2,341 (26)	1,480 (22)	861 (36)	
HF	1,654 (18)	984 (15)	670 (28)	
ASHD	987 (11)	627 (9.4)	360 (15)	
Dysrhythmia	59 (0.65)	37 (0.55)	22 (0.92)	
CVA/TIA	238 (2.6)	143 (2.1)	95 (4.0)	
PVD	244 (2.7)	146 (2.2)	98 (4.1)	
COPD	152 (1.7)	91 (1.4)	57 (2.4)	
Liver Disease	41 (0.45)	28 (0.42)	13 (0.54)	
Cancer	224 (2.5)	179 (2.7)	45 (1.9)	
Median follow-up time**, days [IQR]	537 [166 – 1101]	584 [171 – 1185]	437 [155 – 870]	< 0.001
Median time from diagnosis to start of RRT, days [IQR]	13 [0 – 209]	3 [0 – 20]	488 [309 – 808]	< 0.001
History of kidney transplantation	1101 (12)	764 (11.4)	337 (14)	< 0.001
Total number of deaths	2,821 (31)	2,268 (34)	553 (23)	< 0.001

ED: early diagnosis; LD: late diagnosis; ASHD: atherosclerotic heart disease; COPD: chronic obstructive pulmonary disease; CVA: cerebrovascular accident; DM: diabetes mellitus; GN: glomerulonephritis; HF: heart failure; HTN: hypertension; PVD: peripheral vascular disease; TIA: transient ischemic attack.

*Until April 6^th^, 2019.

**Follow-up time is a time from initiation or RRT until death or April 6^th^, 2019.

The most prevalent age group in the RRT cohort is 45-64, with a median age ranging from 51 to 53 across all groups, with a higher proportion of males (56% vs 44%). The most prevalent ethnicity is Kazakh, representing 64% of RRT patients. Russian ethnicity is the second most prevalent, accounting for 17% of RRT patients.

The most common comorbidity is Hypertension (HTN), present in almost half of RRT patients (47%). Other common comorbidities include diabetes mellitus (DM) (21%) and cardiovascular disease (CVD) (26%). Less common comorbidities, such as liver disease and dysrhythmia, are present in less than 1% of patients. Among RRT patients, the ED group exhibits a higher prevalence of all comorbidities.

The median (IQR) follow-up time from initiation of RRT to death or April 6th, 2019, was 537 (166–1101) days for RRT patients. The median (IQR) time from the first diagnosis to initiation of RRT was 13 days for the overall cohort, particularly 3 days for LD patients and 488 (309–808) days for ED patients. The ED group had a slightly higher percentage of patients receiving transplantation (14% vs 11.4%). The total number of deaths among RRT patients was 2,821 (31%). Lately diagnosed patients accounted for 2,268 deaths (34%), while the second RRT group had 533 deaths (23%).

#### Survival analysis

**Figure 2.**
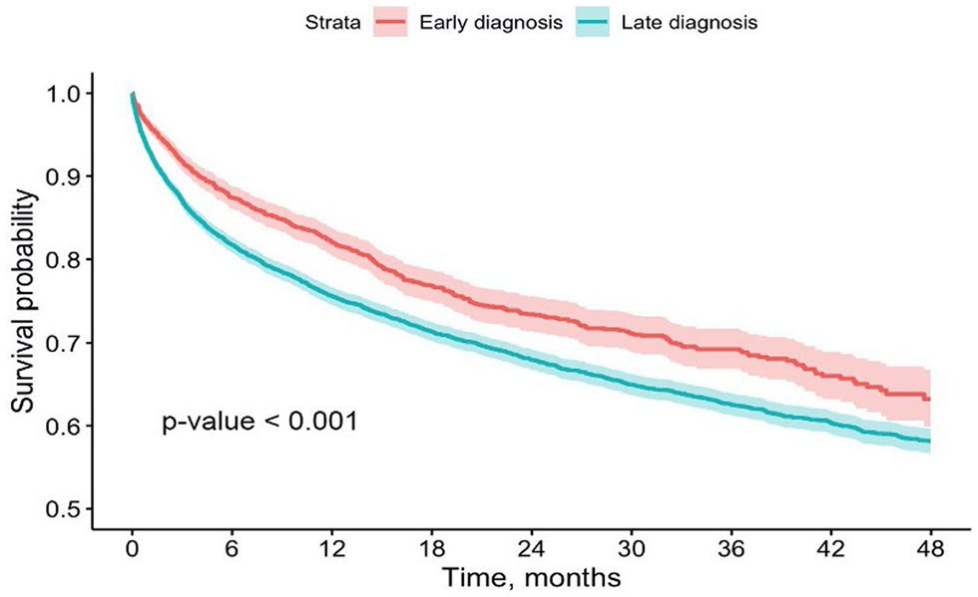
Kaplan–Meier plot demonstrating difference in survival rate between patients from LD and ED groups.

Univariate survival analysis between LD and ED patients is illustrated in Kaplan-Meier plot (Figure 2). Survival probability is significantly higher in patients that starter RRT ≥ 6 months after the diagnosis (*p* < 0.0001). Supplementary Table 2 demonstrates survival probability at certain time points with values taken from the Kaplan–Meier plot (Figure 2).

**Figure 3.**
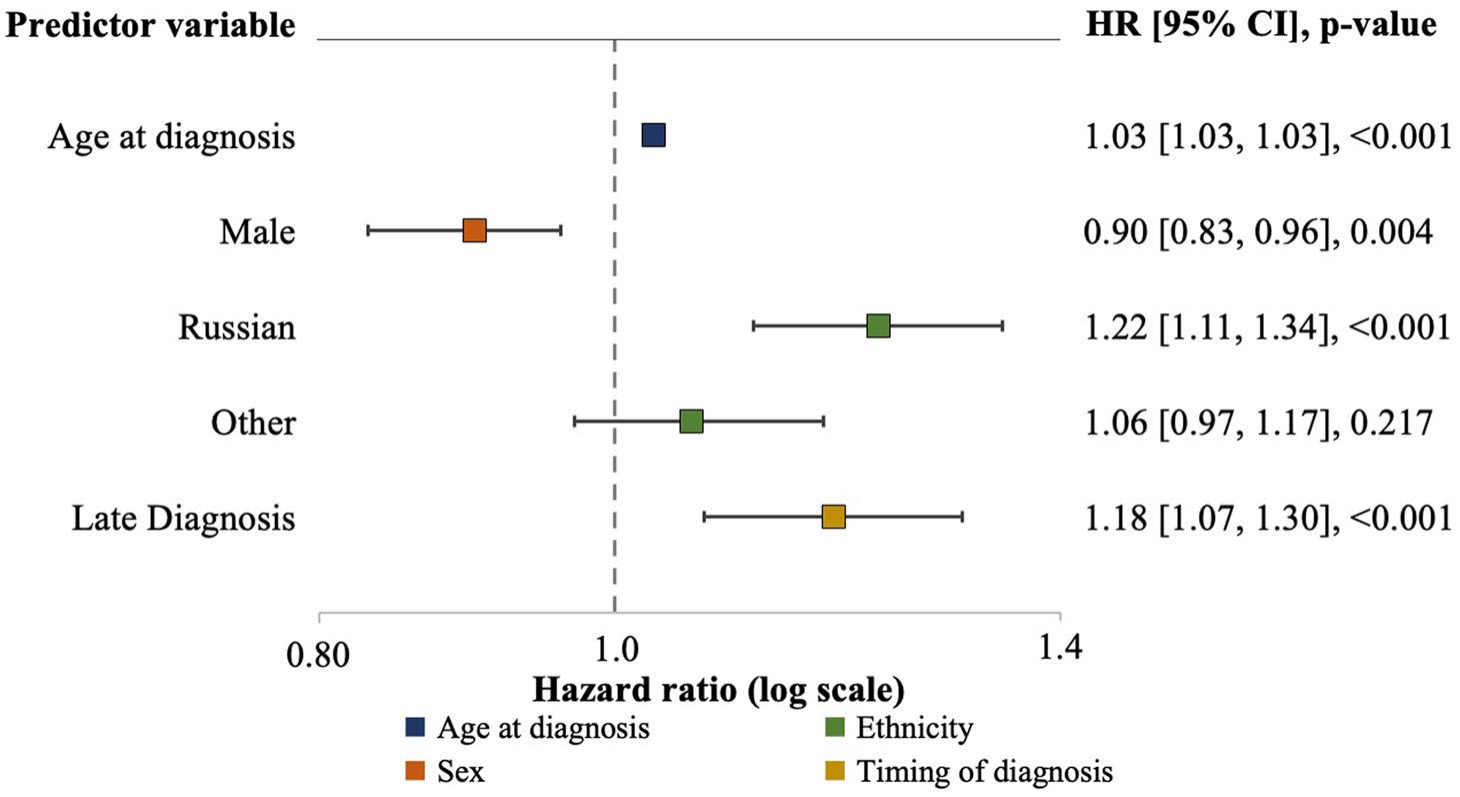
Forest plot demonstrating results of Cox proportional hazards model evaluating factors affecting survival of ESRD patients after commencing RRT.

The results of the multivariate survival analysis using the Cox Proportional Hazards Model are presented in the forest plot (Figure 3). Each additional year at the time of diagnosis contributes to a 3% increase in the risk of death (HR = 1.03, *p* < 0.001). Male patients exhibit a better survival prognosis compared to female patients, with an HR of 0.90 (95% CI 0.83-0.96, *p* = 0.004). Russian ethnicity is associated with a poorer prognosis in comparison to patients with Kazakh ethnicity, indicated by an HR of 1.22 (95% CI 1.11–1.34, *p* < 0.001). However, other ethnicities do not significantly differ in survival prognosis compared to Kazakh ethnicity, with an HR of 1.06 (95% CI 0.97–1.17, *p* = 0.217).

Patients with LD demonstrate a worse survival prognosis with an HR of 1.18 (95% CI 1.07–1.30, *p* < 0.001) compared to ED patients. Comorbidity score and history of transplantation were also included in the Cox regression but are not illustrated as a part of the forest plot. Each point increase in the comorbidity score positively impacts survival prognosis by 2% (HR = 0.98, 95% CI 0.95–1.00, *p* = 0.052). Notably, having a history of transplantation significantly reduces the risk of death by 81% (HR = 0.19, 95% CI 0.15–0.25, *p* < 0.001).

## Discussion

In this retrospective cohort study of 9,097 patients with chronic kidney disease (CKD) in Kazakhstan, those who initiated renal replacement therapy (RRT) with a late diagnosis (LD) had an 18% increased risk of dying compared to those with an early diagnosis (ED). The study cohort predominantly comprised individuals aged over 45, predominantly male, and of Kazakh ethnicity.

The prevalent comorbidities and or potential causes of CKD patients in our study were HTN, DM, and CVD, notably HF. It is crucial to note that our data does not imply a causal relationship between CKD and these comorbidities but rather reports their prevalence in the CKD population. In our cohort, 45% had HTN, while only 11% had DM, indicating potential underdiagnosis, as other studies reported higher proportions. For instance, Bikbov et al. [1] found DM as a cause of CKD in approximately 30.7% of cases, and the USRDS Annual Data Report indicated a 48.3% prevalence of DM among all CKD patients [24]. The observed lower prevalence of CVD, COPD, liver disease, and cancer in our data further highlights the potential underreporting of both CKD and associated comorbidities. Notably, the ED group exhibited a higher prevalence of comorbidities, suggesting this group received a higher degree of medical attention and potentially explaining earlier CKD diagnosis in patients with more comorbidities. Comorbidities themselves demonstrate 2% better survival for each additional score point (*p* = 0.052). Although the impact is not very significant, this could be explained by the fact that as more comorbidities are registered in the electronic health system, more medical attention is potentially provided not only regarding CKD but also regarding other comorbidities, which can affect overall survival. Patients with existing comorbidities but not having them registered in the electronic system can potentially have their comorbid conditions addressed to a lesser degree.

Our study revealed that 74% of CKD patients in Kazakhstan fall into the LD category, surpassing the 71.6% reported by Baer et al. [6]. Furthermore, over 50% of patients initiating RRT were diagnosed just 13 days before treatment initiation, indicating a substantial proportion of ultra-late presentations. Survival analysis supported earlier findings, demonstrating better outcomes for ED patients compared to the LD population. The Kaplan–Meier plot illustrated a survival benefit for ED patients right from the initiation of RRT, with a notable 6% difference in the first 6 months. Cox regression further confirmed the more favorable survival prognosis for the ED group, emphasizing the clear benefit of early CKD diagnosis in Kazakhstan. As previously stated, according to the Kazakhstani protocol for CKD treatment, only individuals with comorbid conditions or symptoms indicative of CKD undergo diagnostic assessment for the disease [19]. However, in 2020, the Ministry of Health implemented new regulations concerning the screening of individuals residing in rural areas aged between 18 and 70 years [25]. This specific group undergoes screening for early detection of kidney diseases at an interval of one year.

Kidney transplantation, known for its superior survival probability and improved quality of life, showed significant advantages among ESRD patients in Kazakhstan, as reflected in our results. Thus, there’s a critical need for more comprehensive identification of CKD patients in Kazakhstan, especially those comorbid with HTN and DM. Early identification offers an opportunity to slow down CKD progression, better prepare patients for RRT, and improve survival outcomes. Study strengths include a large study population and access to the entire Kazakhstani population registered in the UNEHS. However, limitations include the lack of clinical or laboratory data and constraints associated with the databases used. Future efforts should focus on more accurate data input, including CKD stage identification, and leveraging text mining algorithms to extract clinical and laboratory information. This approach could lead to a more thorough analysis and enhanced understanding of CKD and RRT patients in Kazakhstan.

Clinical implications involve the potential implementation of screening methods for patients at risk of CKD, such as those with HTN, DM, and GN. Educating primary care physicians about CKD and appropriate referral timing is essential, aiming to achieve lower levels of late referral worldwide. Nephrologists need to be aware of current practices for transitioning patients to RRT, providing comprehensive patient education on available RRT methods, ESRD consequences, and their impact on patients’ lives.

Several limitations should be discussed. Firstly, the database exhibits notable gaps in essential clinical and laboratory data pertaining to patients. Specifically, there is a lack of information about levels of creatinine, including creatinine levels over a period of time, introducing a potential bias where patients with AKI could be mislabeled as having CKD. We also do not know creatinine levels at the moment of CKD diagnosis, the degree of proteinuria, or the estimation of the severity of any existing comorbidities, such as DM. There is also no available information about the medications that patients were taking or specifications about the type of dialysis they had (hemodialysis vs peritoneal dialysis), although the majority of patients in Kazakhstan undergo hemodialysis. Moreover, the potential for errors during disease coding further compounds these limitations. In addition, the absence of data concerning the cause of mortality restricts the analysis to all-cause mortality metrics only. Despite these acknowledged limitations, the assessment of survival prognosis among patients following the initiation of RRT offers several advantages. First, it serves as an indicator of healthcare efficacy within the nation. The comprehensive nature of the database, encompassing all relevant cases nationwide, facilitates understanding of the disease burden and gives invaluable insights for health policymakers. Furthermore, these findings can inform the development of refined protocols and management strategies within healthcare settings, taking into account sociodemographic variables and cultural nuances. Additionally, they have the potential to improve population-wide awareness initiatives and accentuate the importance of adopting healthy lifestyle practices to mitigate the risk of ESRD. Lastly, the findings may initiate further investigations into the accessibility and affordability of kidney disease treatments.

## Conclusion

In summary, this study unveils crucial aspects of chronic kidney disease in Kazakhstan, revealing a substantial burden with 211,655 identified patients, though suggesting potential underdiagnosis. The prevalence of comorbidities, notably hypertension and diabetes, underscores the need for heightened awareness and screening practices. Strikingly, a majority of patients are diagnosed late, emphasizing the imperative of early detection for improved survival outcomes. Kidney transplantation emerged as a favorable intervention. Regional variations in survival probabilities call for collaborative efforts and knowledge exchange. Despite limitations, this study emphasizes the urgency of enhancing CKD identification, refining data collection, and promoting informed practices among healthcare providers to positively impact patient outcomes in Kazakhstan.

## Supplementary Material

STROBE checklist.docx

Supplementary materials.docx

Figure 1.jpg

Figure 2.jpg

Figure 3 new.jpg

## Data Availability

The data that support the findings of this study are available from the Republican Center for Electronic Health of the Ministry of Health of the Republic of Kazakhstan but restrictions apply to the availability of these data, which were used under license for the current study, and so are not publicly available. Data are however available from the corresponding author, Gaipov A., upon reasonable request and with permission of the Ministry of Health of the Republic of Kazakhstan.
