# Supplementary material for: Late diagnosis of CKD and associated survival after initiation of renal replacement therapy in Kazakhstan: analysis of nationwide electronic healthcare registry 2014–2019
Source: Ren Fail. 2024 Sep 4;46(2):2398182. doi: 10.1080/0886022X.2024.2398182 (PMC11376288; doi:10.1080/0886022X.2024.2398182)
Supplement: Supplementary materials.docx [file IRNF_A_2398182_SM3855.docx]

**SUPPLEMENTARY MATERIALS**

Supplementary Table 1. ICD-9 CM Procedure codes and ICD-10 CM Diagnosis codes used. Codes that start with “^” mean that all codes that start with these characters are utilized. For example, ^N18 includes N18.1, N18.2, N18.3, etc.

| **CKD Diagnosis** | **ICD-9 CM Procedure code / ICD-10 CM Diagnosis code** |
| --- | --- |
| CKD | ICD-10: A18.11, A52.75, B52.0, D59.3, ^E08.2, ^E09.2, ^E10.2, ^E11.2, ^E13.2, ^I12, ^I13, K76.7, ^M10.3, M32.14, M32.15, M35.04, ^N00, ^N01, ^N02, ^N03, ^N04, ^N05, ^N06, ^N07, N08, N11.0, ^N13, ^N14, N15.0, N15.8, N15.9, N16, ^N18, N19, ^N25, ^N26, ^N27, N28.81, N28.82, N28.83, N28.89, N28.9, N31.2, N31.9, ^O10.2, ^O10.3, ^O12.1, ^O12.2, ^O26.83, Q27.1, ^Q60, Q61.00, Q61.02, ^Q61.1, Q62.0, ^Q62.1, Q62.2, ^Q62.3, Q62.4, Q62.5, ^Q62.6, ^Q63, ^Q64, Q79.4, Q79.51, Q80.0, R94.4, ^T86.1, Y84.1, Z48.22, ^Z49, Z94.0, Z99.2 |
| DM | ICD-10: ^E8, ^E9, ^E10, ^E11, ^E13 |
| HTN | ICD-10: ^H35.03, ^I10, ^I11, ^I12, ^I13, ^I15, ^I16, I67.4 |
| GN | ICD-10: D59.3, M31.0, M31.31, M31.7, M32.14, ^N00, ^N01, ^N02, ^N03, ^N04, ^N05, ^N06, ^N07, N08, ^O12.1, ^O12.2, ^O26.83, Z87.441 |
| Cystic disease | ICD-10: N28.1, N28.84, N28.85, N28.86, ^Q61.0, ^Q61.1, ^Q61.2, Q61.3, Q61.5, Q61.8, Q61.9 |
| Dialysis | ICD-10: ^Z49, Z99.2  ICD-9: ^38.95, ^39.27, ^39.42, ^39.43, ^39.93, ^39.94, ^39.95, 54.98, ^86.07 |
| Transplantation | ICD-10: ^T86.1, Z48.22, Z94.0  ICD-9: ^55.6 |
| HF | ICD-10: I09.81, I11.0, I13.0, I13.2, ^I42, I43, ^I50, Z48.21, Z48.280, Z94.1, Z94.3 |
| ASHD | ICD-10: ^I20, ^I21, ^I22, ^I23, ^I24, ^I25, I51.0, I51.2, Z95.1, Z95.5, Z98.61 |
| Dysrhythmia | ICD-10: ^I44, ^I45, ^I46, ^I47, ^I48, ^I49, R0.01, Z45.010, Z45.018, Z45.02, Z45.09, Z95.0, Z95.810, Z95.818, Z95.9 |
| CVA/TIA | ICD-10: ^G45, ^G46, ^I60, ^I61, ^I62, ^I63, ^I65, ^I66, ^I67, ^I68, ^I69 |
| PVD | ICD-10: ^E10.5, ^E11.5, I67.0, ^I70, ^I71, ^I72, ^I73, ^I74, ^I77, ^I79, ^I80, I81, ^I82, I96, ^K55, M31.8, M31.9 |
| COPD | ICD-10: ^J41, J42, ^J43, ^J44, ^J45, ^J47 |
| Liver disease | ICD-10: B251, ^K70, ^K71, ^K72, ^K73, ^K74, ^K75, ^K76, K77, Z48.23, Z94.4 |
| Cancer | ICD-10: ^C, D45, D47.1, D47.9, D47.Z9 |

Supplementary Table 2. Survival data demonstrating survival probability (from Kaplan-Meier plot) at multiple time points after initiation of RRT between LD and ED groups.

|  | **6 months** | **1 year** | **2 years** | **3 years** | **4 years** |
| --- | --- | --- | --- | --- | --- |
| Late diagnosis | 81.7% | 75.5% | 68.0% | 62.5% | 58.2% |
| Early diagnosis | 87.4% | 82.0% | 73.4% | 69.2% | 63.2% |
| p-value | < 0.001 | < 0.001 | < 0.001 | < 0.001 | < 0.001 |
