# Supplementary figures and images for: Late diagnosis of CKD and associated survival after initiation of renal replacement therapy in Kazakhstan: analysis of nationwide electronic healthcare registry 2014–2019
Source: Ren Fail. 2024 Sep 4;46(2):2398182. doi: 10.1080/0886022X.2024.2398182 (PMC11376288; doi:10.1080/0886022X.2024.2398182)

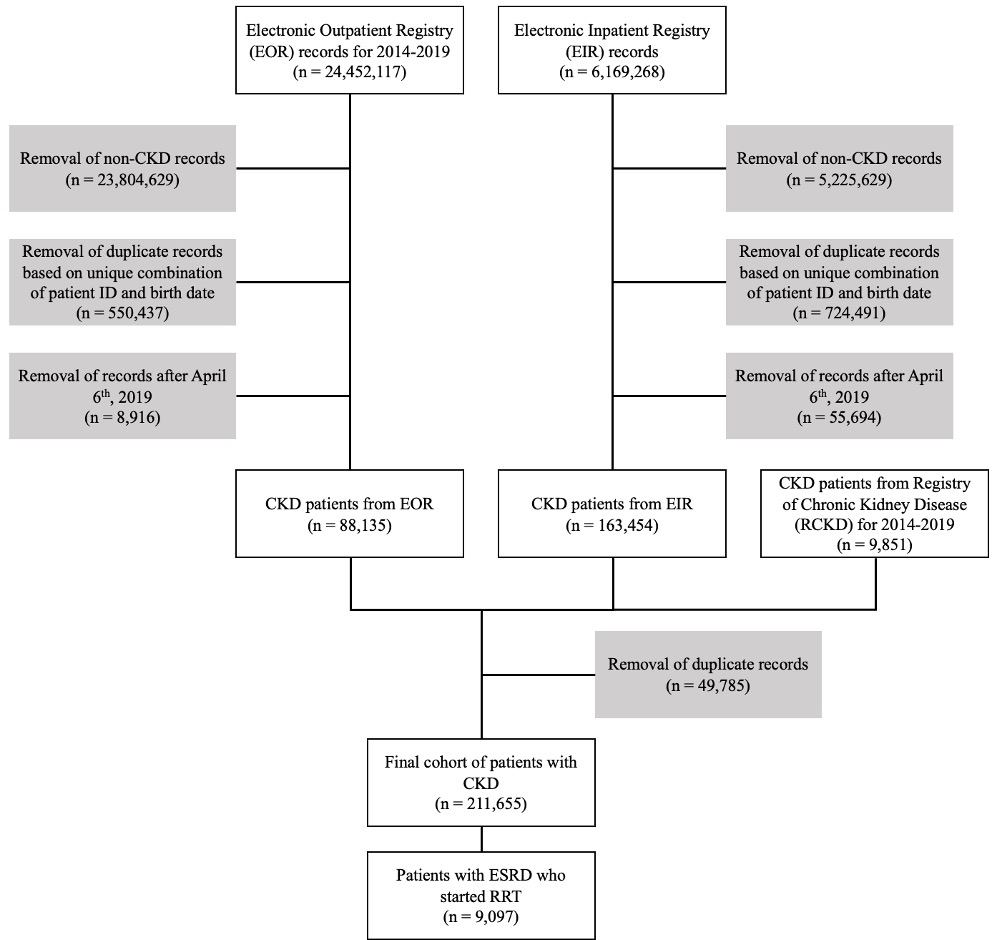

Supplement: Figure 1.jpg [file IRNF_A_2398182_SM3854.jpg]

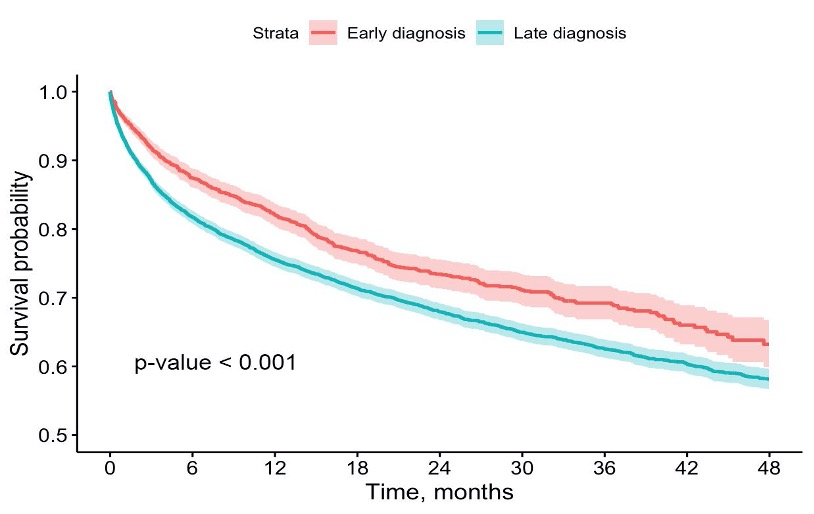

Supplement: Figure 2.jpg [file IRNF_A_2398182_SM3853.jpg]

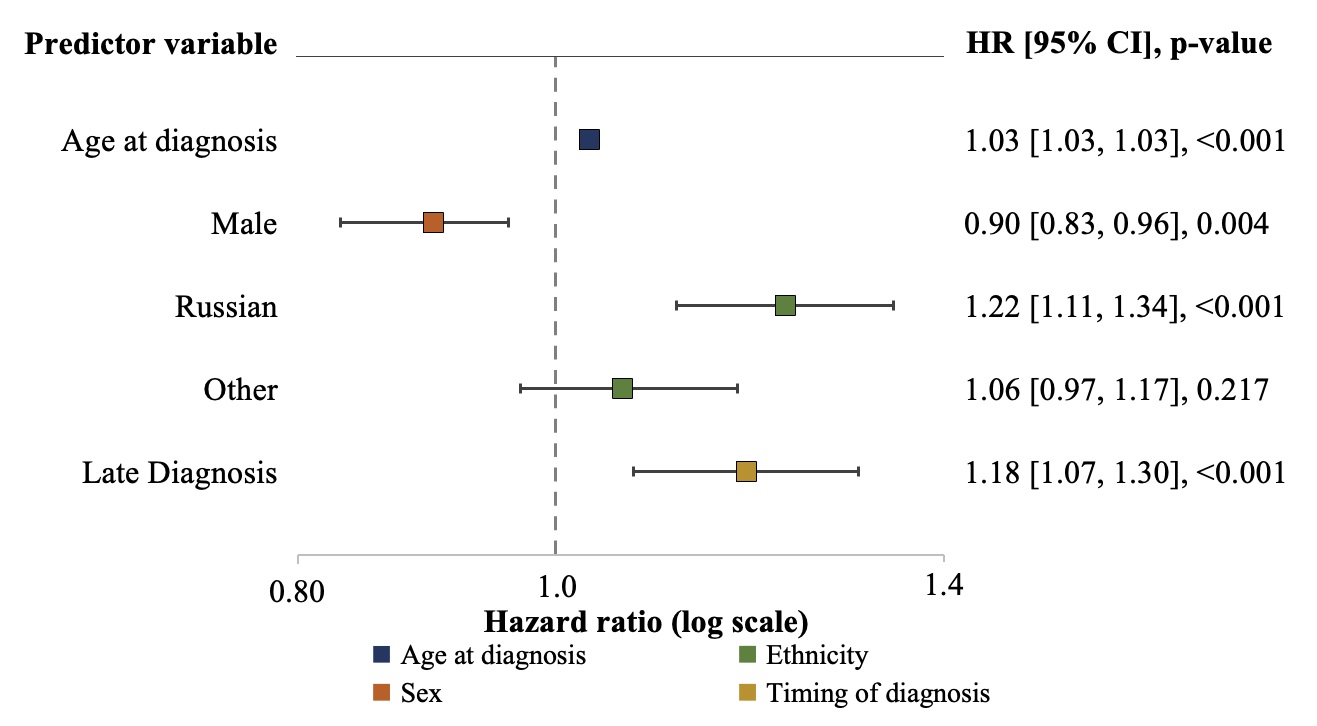

Supplement: Figure 3 new.jpg [file IRNF_A_2398182_SM3852.jpg]
